# Potential Benefits of Berberine in the Management of Perimenopausal Syndrome

**DOI:** 10.1155/2015/723093

**Published:** 2015-02-17

**Authors:** Cristiana Caliceti, Paola Rizzo, Arrigo Francesco Giuseppe Cicero

**Affiliations:** ^1^Department of Medical Sciences, University of Ferrara, Ferrara, Italy; ^2^Laboratory for Technologies of Advanced Therapies (LTTA), University of Ferrara, Ferrara, Italy; ^3^Department of Medicine and Surgery Sciences, University of Bologna, Bologna, Italy

## Abstract

Cardiovascular diseases are one of the leading causes of morbidity and mortality in women after menopause and 56% of all causes of death in Western European countries. Nowadays, with increasing life span, women spend approximately one-third of their life-time in postmenopausal state; therefore, the development of new strategies to improve the prevention and treatment of menopause-associated pathologies is important topic in clinical practice. The studies to assess the safety of hormone replacement therapy in women with estrogen deficiency have not been conclusive due to the relative contraindications; therefore, hormone replacement therapy is prescribed only in selected cases and for a limited time. For this reason, today women are encouraged to use naturally available compounds to prevent or to attenuate menopausal symptoms and correlated pathologies, with fewer side effects. Among these compounds, berberine, an isoquinoline alkaloid derived from plants of the generis *Berberis*, has been recognized as being capable of decreasing oxidative stress, LDL, triglycerides, and insulin resistance and of improving the mood. This review describes the cellular and clinical effects associated with the use of berberine, which suggest that this molecule could be an effective natural supplement to ensure a smooth peri- and postmenopausal transition.

## 1. Introduction

Cardiovascular diseases are one of the leading causes of morbidity and mortality in women after menopause and 56% of all causes of death in Western European countries [1]. Menopause is characterized by an altered hormonal status and a subsequent decrease in life quality affecting each woman differently. Specifically, the decline and eventual cessation of estrogen production are associated with the appearance of uncomfortable symptoms (hot flashes, night sweats, breast tenderness, vaginal dryness, irregular menses, mood changes, and vaginal atrophy) as well as pathologies such as osteoporosis, heart disease, hypercholesterolemia, endothelial dysfunction, vascular inflammation, hyperglycemia, and depression [2]. Currently, women may expect to spend more than a third of their lives after menopause; therefore, the understanding of menopause-associated pathologies and the development of new strategies to improve the treatment of menopause-associated symptoms are important topics in the clinical practice. Results from different studies indicate that the use of hormone replacement therapy (HRT) in menopause needs to be carefully assessed and risks and benefits of the therapy should be evaluated by the clinician for each individual woman. Clearly, the possible problems associated with HRT in a subpopulation in women may be attenuated by other molecules, safer than estrogens, as well as by lifestyle modifications, such as diet, food supplements, and exercise [3]. In recent years, there has been renewed interest in the potential of purified natural products to provide health and medical benefits and to prevent disease. Antioxidant compounds have been shown to be of great benefit in women experiencing menopausal symptoms [4].

Berberine (BBR), a quaternary ammonium salt from the protoberberine group of isoquinoline alkaloids (5,6-dihydrodibenzoquinolizinium derivative) found in the bark, rhizomes, roots, and stems of* Berberis vulgaris* L. (Berberidaceae), exhibits many different types of biological activities [5]. Among them, the best characterized ones are antioxidant, anti-inflammatory, cholesterol-lowering, antihyperglycemic, and antidepressive effects.

This paper will discuss the studies that have shown the potential benefits of berberine supplementation for the reduction, at least partially, of menopause-associated pathologies (oxidative stress, inflammation and hypercholesterolemia-related cardiovascular diseases, hyperglycemia-related diabetes mellitus type 2, and depression) and for the improvement of quality of life in menopausal women.

## 2. Role of Berberine in Oxidative Stress

Estrogen deficiency increases cardiovascular risk, as a result of atherogenic modifications of plasma lipid profile, activation of the renin-angiotensin system, and overproduction of reactive oxygen species (ROS) which quench nitric oxide (NO) [6, 7]. Overproduction of ROS has been shown in pathological situations and can be highly injurious to adjacent structures in cells, including lipid membranes, DNA, and proteins [8]. Increased oxidative stress and reduced NO bioavailability are important contributing factors of menopause-related endothelial dysfunction, atherosclerosis, hypertension, cardiovascular, and renal diseases [9, 10].

Impaired vascular function due to ROS overproduction which occurs during estrogen deficiency could be normalized by HRT in women and animal models of menopause [11, 12]. In fact, following the onset of either permanent or transient estrogens deprivation, oxidative stress increases significantly [4]. Serum concentrations of inflammatory cytokines and prooxidant biomarkers such as glutathione, 4-hydroxynonenal, and malondialdehyde were found to be higher in postmenopausal than in premenopausal women [13]. The elevation of cytokines and prooxidant makers suggests that there is a high degree of oxidative stress in the postmenopausal state [13, 14].

One of the major sources of ROS production in cells is a family of membrane-associated enzymatic complexes called nicotinamide adenine dinucleotide phosphate (NADPH) oxidase (NOX) [15]; its activation is often associated with high levels of fatty acids, cholesterol, glucose, or advanced glycation end products (AGEs), all linked to menopausal state [16–18]. Among various NOX isoforms, BBR was reported to suppress the overexpression of NOX 2, 4 and to decrease ROS production in macrophages and endothelial cells upon stimulation with inflammatory stimuli [19, 20]. In endothelial cells, BBR attenuates LDL oxidation induced by ROS and reduces the collapse of mitochondrial membrane potential, the chromosome condensation, the cytochrome C release, and the caspase-3 activation [21]. Circulating endothelial microparticles (EMPs), vesicular structures found in plasma from patients with vascular diseases so utilized as a surrogate marker of endothelial dysfunction, are oxidative stress inducers; they promote upregulation of NOX4 expression and ROS production. It has been reported that BBR reversed NOX4-derived ROS production in human umbilical vein endothelial cells (HUVECs) [22].

NOX could be negatively regulated by adenosine monophosphate-activated protein kinase (AMPK) activation [23, 24]; in fact AMPK activators, such as metformin, may exert their cardiovascular protective function through NOX inhibition [25]. AMPK pathway is activated by BBR [26] and it seems to play a pivotal role in mediating its antioxidant activity [27, 28].

AMPK plays also an important role in regulating function of NO synthesis in endothelial cells. In fact, AMPK is an upstream kinase of endothelial nitric oxide synthase (eNOS) which promotes the phosphorylation of eNOS at Ser1177 site as well as the formation of eNOS and HSP90 complex and NO production [9, 29]. Menopause-related decrease in estrogen receptors correlates with lower eNOS expression and activation [30] in both animal and human models of menopause [7, 12] as eNOS activity is notably regulated by estrogens through the modulation of the eNOS/caveolin-1 (Cav-1) complex formation [31]. Zhang and colleagues observed that in HUVECs BBR ameliorates palmitate-induced endothelial dysfunction by upregulating eNOS and downregulating NOX4 through the activation of AMPK [32]. In both cultured endothelial cells and blood vessels isolated from rat aorta BBR enhanced eNOS phosphorylation and attenuated high glucose-induced generation of ROS, cellular apoptosis, NF-*κ*B activation, and expression of adhesion molecules through AMPK signaling cascade activation, a key event in preventing oxidative and inflammatory signaling [33].

Besides NADPH oxidase downregulation and NO production, AMPK activation has been linked to upregulation of the antioxidant enzyme superoxide dismutase (SOD) [34, 35], which induces the dismutation of anion superoxide in hydrogen peroxide. An increased SOD expression in BBR treated diabetic mice was observed [36, 37]. Glutathione (GSH) is another antioxidant enzyme which helps to maintain the balance of redox state in organisms and it is a substrate of glutathione peroxidase (GSH-Px) in the clearance of peroxides [38]. BBR treatment promotes a GSH-Px and SOD hyperactivation in the liver of mice [39], attenuates H_2_O_2_-induced ROS production, and increases detoxifying enzymes GSH-Px and SOD in NSC34 motor neuron-like cells [40].

## 3. Role of Berberine in Cardiovascular Disease Risk

The increased cardiovascular disease risk at the menopause is associated with decreased ovarian function [41, 42] and is in part due to arterial dysfunction and a less favorable blood lipid profile. Exogenous estrogen and progesterone, in the form of HRT, have been shown to reduce plasma concentrations of LDL cholesterol and increase concentrations of HDL cholesterol [43] even if their use in menopause is still object of debate because of the increased risk of breast cancer, ictus, ischaemic cardiomyopathy, and thrombus detachment [44, 45].

High levels of low-density lipoprotein (LDL) and their oxidized counterpart, oxidized LDL (oxLDL), in the blood vessels represent a major risk factor for endothelial dysfunction and atherosclerosis [46]. Inactivity of LDL receptor (LDLR) or its low-level expression initiates accumulation of LDL in blood vessels [47]. On the other hand, the receptor of oxLDL, lectin-like oxidized low-density lipoprotein receptor-1 (LOX-1) identified as the main endothelial receptor for oxLDL and also present in macrophages and smooth muscle cells (SMC), activates a proatherogenic cascade by inducing endothelial dysfunction, SMC proliferation, apoptosis, and the transformation of macrophages into foam cells and platelet activation via NF-*κ*B activation [48]. LOX-1 contains a lectin-like extracellular C-terminal domain which interacts with oxLDL, proteolytically cleaved and released as a soluble circulating form (sLOX-1) that reflects the increased expression of membrane-bound receptors and disease activities [49].

Proinflammatory and oxidative stimuli related to atherogenesis [48] such as TNF*α* or interferon *γ* promote the oxLDL formation which in turn develops a vicious cycle by the activation of LOX-1 linked to NF-*κ*B to promote transcription of proinflammatory molecules [50]. In arterial walls oxidative stress and inflammation are closely linked; LOX-1 is undetectable in healthy vessels but overexpressed in atherosclerotic lesions and in acute coronary syndromes [51]. Thus circulating sLOX-1 could be a potential cardiovascular disease biomarker [49].

It has been reported that in cultured placental cells a combination of ethinyl estradiol and desogestrel increases the expression and functional activity of LDLR and decreased the expression of LOX-1 [52–54].

BBR elevates LDLR expression in human liver cells [55] through ERK activation with a sterol regulatory element binding proteins (SRPB) independent mechanism [56, 57]. In another report it has been reported that BBR induces proprotein convertase subtilisin/kexin type 9 (PCSK9), an enzyme that posttranscriptionally upregulates LDLR and SREBP-2 [58]. In accordance with previous cited studies, it has been showed that in rat livers a combination of BBR with simvastatin increased the LDLR gene expression to a level significantly higher than that in monotherapies [59].

In human macrophage-derived foam cells treated with oxLDL, BBR inhibits the expression of LOX-1 [60, 61] as well as the oxLDL uptake of macrophages and reduces foam cell formation in a dose-dependent manner [61] by activating the AMPK-SIRT1-PPAR*γ* pathway [62]. Chi and colleagues [63] demonstrated that BBR combined with atorvastatin is more effective in diminishing LOX-1 expression than atorvastatin alone in monocyte-derived macrophages both* in vitro* and* in vivo* in rats through modulation of endothelin-1 receptor [62]. It is still unknown whether BBR could affect LOX-1 expression in endothelial cells.

BBR improves also the survival of TNF*α*-treated endothelial progenitor cells (EPCs) via the activation of PI3K/AKT/eNOS signaling pathway [64] possibly through AMPK activation. Wu and colleagues showed, both* in vitro* and* in vivo*, that BBR reduces the leukocyte-endothelium adhesion and vascular cell adhesion molecule-1 (VCAM-1) expression induced by lipopolysaccharide (LPS). BBR was further confirmed to inhibit the nuclear translocation and DNA binding activity of LPS-activated NF-*κ*B signaling pathway [65].

The lipid-lowering activity of BBR, alone or in association with other nutraceuticals, has been clearly confirmed in a relatively large number of randomized clinical trials, involving a large part of women (usually a half, almost all in peri- or postmenopausal age) [66]. In a large placebo-controlled, randomized clinical trial, it has been reported that short-term consumption of a combined nutraceutical containing isoflavones and BBR out on 120 mild dyslipidemic postmenopausal women significantly lowered plasma total cholesterol (13.5% ± 0.7 versus 0.2% ± 0.5), LDL cholesterol (12.4% ± 1.5 versus 0.8% ± 0.7), and TG (18.9% ± 2.5 versus 1.3% ± 1.2) improving menopausal symptoms compared with placebo [67].

In a subsample of the same study, it is shown that the assumption of isoflavones and BBR also improved the serum levels of matrix metalloproteinases, known to promote the invasion of inflammatory cells by degrading the extracellular matrix [2].

The anti-inflammatory effect of BBR was also confirmed in patients with acute coronary syndrome following percutaneous coronary intervention [68].

Thus, BBR seems to be a promising preventive treatment in the initial key steps of atherogenesis.

## 4. Role of Berberine in Diabetes Mellitus Type 2

Different studies have shown that the incidence of diabetes mellitus type 2 (T2DM) is higher among menopausal women [69, 70]. In fact, estrogen influences not only vascular functions but also insulin sensitivity [71]. T2DM is a chronic disease characterized by hyperglycemia and insulin resistance in peripheral tissues, particularly in the liver, muscles, adipocytes, and pancreatic *β*-cells. Individuals with insulin resistance have either decreased levels or absence of insulin receptor expression (InsR) [72–74] and subsequent hyperglycemia. Oxidative stress participates in development and progression of T2DM, in which changes of SOD and catalase (CAT) were noted in T2DM mice [36].

It has been reported that taking a low dose of combined HRT (a combination of estrogens and progesterone) led to a decreased risk of developing diabetes and to better glucose control in postmenopausal women [75]. Morán and colleagues compared the effects of estradiol and genistein treatments on insulin signaling pathway in the cerebral cortex of ovariectomized young and aged female rats. They observed that aging decreases the translocation of the insulin dependent glucose transporter-4 (GLUT4) and 17*β*-estradiol but not genistein which favours GLUT4 translocation [76].

BBR exhibited a high hypoglycemic potential; it has been shown that BBR activates AMPK with subsequent induction of glycolysis [77]. AMPK, as an intracellular energy receptor, has attracted more attention and become a new target for the treatment of diabetes and its cardiovascular complications due to its regulatory effect on endothelial cell function and energy homeostasis. In H9c2 myoblast cell line treated with insulin to induce insulin resistance, BBR attenuated the reduction in glucose consumption and glucose uptake at least in part via stimulation of AMPK activity [78]. BBR enhanced acute insulin-mediated GLUT4 translocation and glucose transport in insulin-resistant myotubes through activation of AMPK and PI3K pathway [79].

Besides the role in AMPK signaling, Kong and colleagues showed that BBR increased insulin receptor (InsR) messenger RNA and protein expression in a variety of human cell lines and hepatitis B virus transfected human liver cells [80]. In a clinical study, the same group observed that BBR significantly lowered fasting blood glucose (FBG), hemoglobin A1c, triglycerides, and insulin levels in patients with T2DM as well as metformin and rosiglitazone (a combination commonly used for the T2DM therapy); the percentages of peripheral blood lymphocytes expressing InsR were significantly elevated after therapy [81].

BBR exhibited similar hypoglycemic potential as glibenclamide (an anti-T2DM drug that stimulates the release of insulin) to lower area under the curve of the fasting blood glucose in the kidney, liver, and brain of mice with T2DM [36].

The dose-dependent antidiabetic properties of BBR have been clearly confirmed in a relatively large number of randomized clinical trials, involving a large number of women (usually 50%, almost all in peri- or postmenopausal age) [82].

Thus, on the basis of the available evidence, we can reasonably conclude that BBR could be an ideal supplementation for T2DM since it acts with a mechanism different from the three drugs commonly utilized in therapy: glibenclamide, metformin, and rosiglitazone.

## 5. Role of Berberine in Depressive Disorder

During the menopausal transition between 15% and 50% of women experience depressive symptoms; in 15% to 30% of perimenopausal women, they are severe enough to be regarded as a depressive disorder. Fluctuations in gonadal hormone levels are thought to contribute to these depressive conditions and HRT is commonly used to alleviate climacteric symptoms [83].

A combination of interactions between neurotransmitters [84], neuropeptides [85], oxidative and nitrosative stress [86], and cytokines [87] are thought to take part in pathogenesis of depression. It is theorized that the additive effect of enhancing neurotransmission in three monoamine systems (serotonin, norepinephrine, and dopamine) may lead to improved efficacy and quicker onset of antidepressant response [88]. Clinical studies have reported that patients with depression presented also oxidative disturbances such as elevated lipid peroxidation products and reduced levels of SOD [89, 90]. NF-*κ*B activity is regulated at least in part by the intensity of intracellular oxidative and nitrosative stress and, in turn, controls the regulation of genes encoding proteins involved in immune and inflammatory responses [91]. Depressed patients often display enhanced cytokine levels including interleukin-6 (IL-6), C-reactive protein, interleukin-1-beta (IL-1*β*), and TNF*α*; they can enter the brain and may cause alterations of the metabolism of serotonin and dopamine [92]. Thus these studies showed a correlation between oxidative and nitrosative stress, increased levels of cytokines, and altered levels of biogenic amines.

Mechanistically, estrogen plays an important role in mood and cognitive regulation [93]. It is reported that monoamine oxidase-A (MAO-A) total distribution volume, an index of MAO-A density, is elevated in perimenopausal women [94].

BBR inhibited the immobility period in mice in both forced swim and tail-suspension test, two animal models of depression, in a dose independent manner [95, 96]. Among the reported bioactivities of BBR there is the inhibition of MAO-A enzyme activity [97]. In fact acute and chronic administration of BBR in mice resulted in increased levels of norepinephrine, serotonin, and dopamine, neurotransmitters induced by MAO-A enzyme [95]. In accordance with Kulkarni and colleague data, Arora and Chopra showed the protective antidepressant-like effect of BBR against the reserpine-induced biogenic amine depletion (a monoamine depletor commonly used to induce depression in animals [98]) and against oxidative nitrosative stress-mediated inflammatory cascade and apoptotic signaling pathway in rats [99].

Recently, the important role of endoplasmic reticulum protein sigma-1 receptors (sigma receptors) in the modulation of various neurotransmitters has been identified. It seems to be a promising target for the pathophysiology of neuropsychiatric disorders, in particular for depression, and sigma-1 receptor modulators are considered the drugs of the future for the treatment of major depression and anxiety. It is reported that BBR has an effect on sigma receptor-1 similar to many synthetic antidepressant drugs [100].

However, at the best of our knowledge, there are no available data on the evaluation of the potential antidepressant effects of BBR in human.

## 6. Berberine Tolerability and Safety

**Figure 1:**
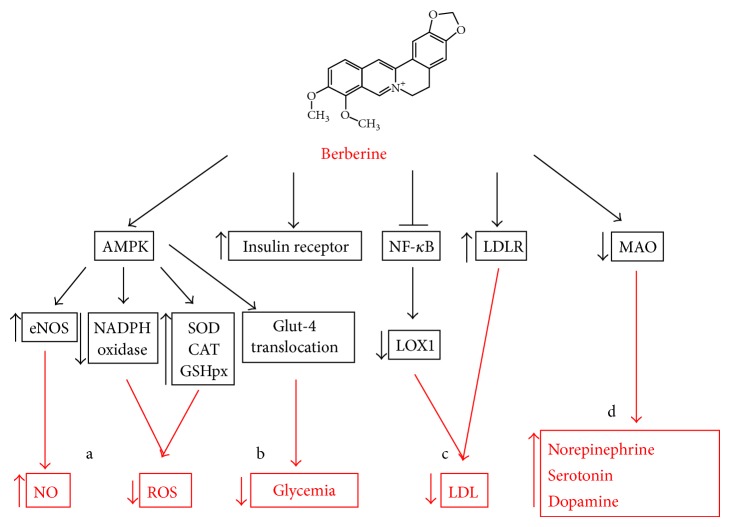
Molecular pathways modulated by BBR. (a) BBR could inhibit oxidative stress through AMPK activation that leads to a downregulation of NADPH oxidase expression and an upregulation of the antioxidant enzymes SOD, CAT, and GSHpx. (b) BBR administration could decrease glycemia through the increase of insulin receptor expression and the AMPK-modulated Glut-4 translocation. (c) BBR could decrease circulating LDL by inhibiting NF-*κ*B modulated LOX-1 expression in endothelial cells and inducing LDLR expression in hepatic cells. (d) BBR could inhibit the expression of MAO, leading to an upregulation of the mood-stabilizers neurotransmitters norepinephrine, serotonin, and dopamine.

**Figure 2:**
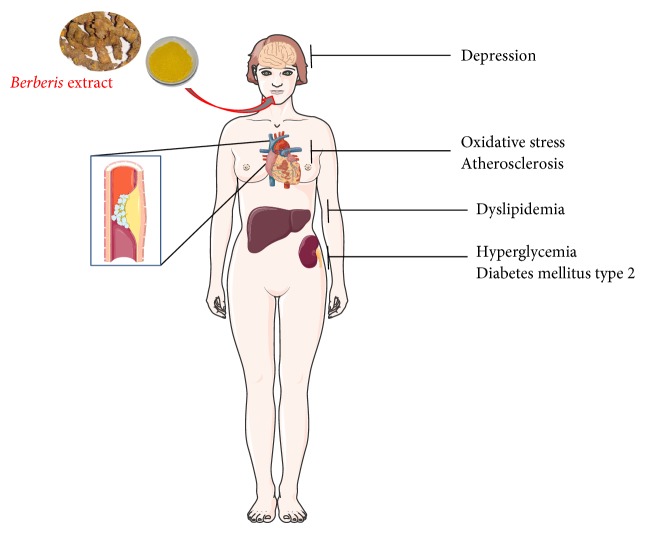
BBR actions and target organs.* Berberis* extract administration could counteract, at least partially, depression, oxidative stress, atherosclerosis, hypercholesterolemia, hyperglycemia, and diabetes mellitus type 2 protecting women from menopause-associated pathologies.

Representative figures to summarize the molecular pathways modulated by BBR and its effects on organs and tissues are shown (Figures 1 and 2). Standard doses of BBR are usually well-tolerated and adverse reactions are rare. On the contrary, high doses have been associated with arterial hypotension, dyspnoea, flu-like symptoms, gastrointestinal discomfort, constipation, and cardiac damage. The LD50 (lethal dose 50) of highly purified formulation of berberine sulfate is 25 mg/kg in mice while the one of* Berberis vulgare* is moderately high (LD50 = 2.6 ± 0.22 g/kg b.w. in mice) [101].

By using sorbitol and breath hydrogen tests it has been shown that BBR delays small intestinal transit time [102]; this may account for part of its gastrointestinal and antidiarrheal side effect [103]. The main mechanism of pharmacological interaction of BBR involves cytochrome CYP3A4 and intestinal P-glycoprotein, the major determinants of bioavailability of orally administered drugs; in renal transplant recipients the coadministration of BBR and cyclosporine A increases the mean cyclosporine A half-life by 2.7 hours [104] thus causing increased cyclosporine A bioavailability and reduced metabolism. BBR is involved in different pharmacological interactions: the drug displaces bilirubin from the albumin about tenfold more than phenylbutazone and also warfarin, thiopental, and tolbutamide from their protein binding sites, increasing their plasma levels [105]. For the cited effects the use of BBR should be avoided in jaundiced infants and pregnant woman, even in small dosage [106].

Although CYP3A4 is the well-known target, BBR also inhibits CYP1A1* in vitro*, therefore potentially interacting with drugs metabolized by this cytochrome isoform as well [107]. The impact of this observation in clinical practice has to be evaluated since some environmental contaminants such as aryl-hydrocarbons are metabolized by CYP1A1 [108].

Lin and colleagues reported that 24 hours of BBR treatment upregulated the multidrug-resistant transporter (pgp-170) expression in oral, gastric, and colon cancer cell lines causing increased cell viability as compared to the effect of the chemotherapeutic agent Paclitaxel. These results suggest that BBR modulates the expression and function of pgp-170 that leads to a reduced response to Paclitaxel in cancer cells [107]. Again, no clinical report of a significant pharmacological interaction is yet available.

Overall there are a large number of recent clinical trials supporting the short-term safe use of this nutraceutical, especially when used at a lipid-lowering dosage.

## 7. Summary

The reduced estrogen level characterizing menopause is associated with the insurgence of discomforts and pathologies which strongly affects the quality of life of many women. There is growing evidence that berberine can, at least partially, minimize the negative consequences on the organism caused by low estrogens levels, without the unwanted side effects associated with commonly prescribed HRT. While the search for a HRT completely free of risks continues, BBR could represent a safe and efficient tool to sustain women during the menopausal transition.
